# I-TEP: A Simple and Affordable Method to Measure Permeability in Reconstructed Tissues Combined with DAMO–TSC-Based Urea Assay

**DOI:** 10.3390/mps9030073

**Published:** 2026-05-03

**Authors:** Yudaï Sahuc, Elissa Elia, Christophe Caneparo, Nathan Félix, Marilou Hardy, Stéphane Chabaud, Stéphane Bolduc

**Affiliations:** 1 Centre de Recherche en Organogénèse Expérimentale/Laboratoire d’Organogenèse Expérimentale (LOEX), Centre Hospitalier Universitaire (CHU) de Québec-Université Laval Research Center (Regenerative Medicine Division), Université Laval, Quebec City, QC G1V 0A6, Canada; yudai.sahuc@crchudequebec.ulaval.ca (Y.S.); elissa.elia@crchudequebec.ulaval.ca (E.E.); marilou.hardy.1@ulaval.ca (M.H.); stephane.chabaud@crchudequebec.ulaval.ca (S.C.); 2 Department of Pediatrics, Gynecology and Obstetrics, Faculty of Medicine, Geneva University Hospitals, University of Geneva, CH-1205 Geneva, Switzerland; 3 Département de Génie Biologique, Institut Universitaire de Technologie (IUT) Lyon1, Université Claude Bernard Lyon 1, 69100 Lyon, France; nathan.felix@gmx.fr; 4 Department of Surgery, Université Laval, Quebec City, QC G1V 0A6, Canada

**Keywords:** permeation test, urea dosage, tissue engineering

## Abstract

Trans-epithelial permeability is a critical functional parameter for reconstructed tissues, particularly in genitourinary tissue engineering, where urine leakage must be avoided. Although Franz diffusion cells are considered the gold standard for permeability measurements, their cost and limited accessibility restrict their widespread use. In parallel, the reliable quantification of urea in culture media remains challenging due to protein interference and assay cost. The Inexpensive Trans-Epithelial Permeability (I-TEP) test is a simple and a low-cost Franz-like permeability system which can be combined with an optimized diacetyl monoxime–thiosemicarbazide (DAMO–TSC) colorimetric urea assay. I-TEP system relies on readily available laboratory components to create physically separate donor and receiver compartments, with the tissue acting as the sole diffusion interface. The DAMO–TSC assay was optimized through systematic evaluation of deproteinization, incubation time, storage conditions, and serum interference. The I-TEP test showed a strong correlation with conventional Franz diffusion cells when testing similar tissue samples. Deproteinization was identified as a mandatory step for accurate urea quantification in serum-containing media. The combined approach was successfully applied on engineered genitourinary tissues, demonstrating sensitivity to tissue maturation and cellular composition. This protocol provides a proof of concept for an affordable, robust, and autonomous method for routine permeability assessment, bridging the gap between costly commercial systems and high-throughput experimental needs.

## 1. Introduction

Trans-epithelial permeability remains a key physiological parameter that needs to be assessed in many fields including pharmacology and cosmetics as well as in research such as cell biology, microbiology and regenerative medicine. Testing permeability is an important checkpoint for assessing the functionality of reconstruct urological tissues before implantation in animals or patients, for example in the case of hypospadias or stenosis correction where urine leakage should be avoided to prevent fibrosis [1,2,3]. Furthermore, these measurements are important for optimizing medicine delivery systems and advancing research as exemplified by neurodegenerative diseases such as Alzheimer’s [4] or oncology [5].

The study of permeability can involve epithelia, synthetic membranes, or biological barriers. Over time, it has progressively been structured around methods that have now become standard. Historically, the foundation of permeability studies lies in the work of Adolf Fick [6] on diffusion laws and that of Overton [7] on cellular permeability to solutes, establishing that lipophilicity promotes diffusion across membranes. Among the earliest experimental models were the Ussing chambers in which the tested sample physically separates two liquid-filled compartments [8]. These chambers allow investigators to measure electrical parameters such as transepithelial resistance. Later, the development of transwell inserts made it possible to physically separate cells—potentially forming monolayers—from another compartment. These inserts can also be coated with matrix molecules to enhance physiological relevance. Various types of molecules, including fluorescent tracers, can be used with these models to assess permeability across different substrates, including epithelial layers.

Franz diffusion cells were introduced in 1975 by Thomas J. Franz [9]. The principle is straightforward: a donor chamber is separated from a heated, stirred receptor chamber by the tissue being evaluated. The system allows sampling from the receptor chamber at regular time intervals. Their industrial spread in the 1980s, followed by adoption by regulatory authorities such as the Food and Drug Administration (FDA) in the 1990s–2000s, established them as a standard method [10]. Modern versions now include precise thermal control, improved materials, and sometimes continuous flux acquisition. Nevertheless, the cost of a complete apparatus may limit accessibility, especially for sporadic use.

To democratize this essential test, an alternative method using similar principles (i.e., separation of a donor and receiver chambers using the surface to test) was elaborated to evaluate same parameters using more affordable materials. For this purpose, a home-made Franz-like system, the I-TEP (Inexpensive Trans-Epithelial Permeability) test, was designed to evaluate permeability parameters similarly to Franz diffusion chamber. The robustness of this system was validated across diverse experiments, including collagen hydrogels and self-assembled genitourinary tissues.

In the case of genitourinary tissue engineering, the permeability of urea through the reconstructed tissue is an absolute requirement to be sure leakage will be avoided. Also, in parallel with the permeation test, we will optimize a dosage of urea in cell culture medium. Urea is one of the earliest biological analytes to be measured in clinical biochemistry, as it is a key marker of renal function and nitrogen metabolism. As a result, many assays have been developed over time.

The DAMO reaction has been under investigation for several years. In the late 60s, the development of manual and automatized methods has been an important technical improvement [11]. Due to many factors like hazardous reagents, low sensitivity and deviance of standard curves, early iterations were questionable. In fact, scientists needed to establish a balance between sensitivity, simplicity and safety. In early 80s, an ultramicro colorimeter capable of measuring picomolar quantities of urea from nanoliter-scale samples has been designed [12]. At the same time, a more sensitive and stable protocol suitable for both deproteinized and with serum samples was developed [13]. They laid the foundations for an optimized DAMO–TSC, focused on robustness and reproducibility. Recently, the chemical reaction was optimized by combining sulfuric acid and phosphoric acid with ferric ionic and thiosemicarbazide [14]. Thus, they obtained a stabilized chromogen reaction considered as an historical photodegradation problem, thereby rehabilitating the DAMO reaction.

Briefly, urea reacts with diacetyl monoxime under strongly acidic conditions (sulfuric acid, H_2_SO_4_) in the presence of an intensifying agent (thiosemicarbazide, TSC) and a catalytic oxidant (Fe^3+^). This combination produces a pink-to-orange chromophore absorbing between 520 and 540 nm. This chemical mechanism does not rely on any enzyme and is therefore not susceptible to enzymatic inhibition (for example by certain antibiotics). Moreover, the final coloration is highly stable thanks to the action of TSC, and the overall cost of the assay is minimal. However, the method requires a heating step (≈95 °C) and the handling of strongly acidic solutions.

For these reasons, a competing method is also widely used: the enzymatic urease assay. Although simple to perform and robust, it is more expensive and can be sensitive to potential inhibitors, which limits its use to specific applications.

The objective of this study was to develop and validate a low-cost, Franz-like permeability system compatible with standard culture plates, together with an optimized DAMO–TSC urea assay suitable for protein-containing media.

## 2. Experimental Design

### 2.1. Ethics Statement

This study was conducted according to the Declaration of Helsinki and was approved by the institution’s committee for the protection of human participants (Comité d’éthique de la recherche du CHU de Québec-Université Laval, protocol number 2012-1341). All patients provided informed written consent prior to biopsies.

### 2.2. Cell Culture

Urethral urothelial cells (UUCs) or urothelial cells (BUCs) were grown on dermal fibroblast feeder cells (foreskin fibroblasts growth arrested with 60 Gy γ-radiation) into a cell-culture-treated flask, T75, in UC medium (DME/F12 (71.25%/23.75%) supplemented with 5% Fetal Clone II (FBS-H), 24.3 µg/mL adenine, 5 µg/mL crystallized bovine insulin, 1.1 µM hydrocortisone, 0.212 µg/mL isoproterenol hydrochloride, 10 ng/mL epidermal growth factor, 100 U/mL penicillin and 25 mg/mL gentamicin) until 70–80% confluency maximum.

UUC and fibroblast cells (UF) were isolated as described previously [15] from human urethral biopsy specimen obtained from male donor submitted to trans surgery aged 22, 23, and 28 years. BUC and bladder fibroblasts (BFs) were isolated from healthy parts of a bladder biopsy from a patient during surgery. Dermal fibroblasts (DFs) were isolated from a human skin biopsy obtained during an esthetic surgery. Cells were stored in liquid nitrogen at −196 °C until utilization.

UFs, BFs or DFs were expanded into a cell-culture-treated flask, T75, (4306410, Corning, Glendale, AZ, USA) in a Dulbecco–Vogt modification of Eagle’s medium for 2 weeks in Fb medium (DME supplemented with 10% Bovine Calf Serum, 100 U/mL penicillin and 25 mg/mL gentamicin) until they reached confluency.

The compositions of the different media are detailed in Tables S1 and S2.

### 2.3. Materials

All materials were used as received.

#### 2.3.1. Tissue Engineering

The materials used for tissue engineering are detailed in the Procedure Section and in several articles (e.g., [16]).

- Six- and twelve-well plates (Falcon, ThermoFischer, Waltham, MA, USA).
- Petri dish.
- Whatman 4 filter paper.
- Air/liquid support (metallic support, custom-made).
- Twicers chirurgical curbed.
- Collagen Type I (5 mg/mL, PureCol EZ gel solution, Sigma-Aldrich, Cat# 5074, St. Louis, MO, USA).
- 2-phospho-L ascorbic acid (Sigma-Aldrich, Cat# 49752, St. Louis, MO, USA).
- Dulbecco–Vogt modification of Eagle’s medium (DME) (Gibco, Cat# 12800-017, Grand Island, NY, USA).
- Bovine Calf Serum (FBS) (Corning, Cat# 35-053-CM, Woodland, CA, USA).
- Ham’s F12 (F12) (Gibco, Cat# 21700-075, Grand Island, NY, USA).
- Fetal Clone II (FBS-H) (Hyclone Cytiva Fetal Clone II, Cat# SH3066.03, Logan, UT, USA).
- Adenine (Sigma-Aldrich, Cat# A2786, St. Louis, MO, USA).
- Crystallized bovine insulin (Sigma-Aldrich, Cat# I5500, St. Louis, MO, USA).
- Hydrocortisone (Galenova, Cat# HY220-0005, Scarborough, QC, Canada).
- Isoproterenol hydrochloride (Sigma-Aldrich, Cat# I5627, St. Louis, MO, USA).
- Epidermal growth factor (Austral Biologicals, Cat# GF-010-8, San Ramon, CA, USA).
- Penicillin (Sigma-Aldrich, Cat# P3032, St. Louis, MO, USA).
- Gentamicin (MP Biomedicals, Cat# 190057, Solon, OM, USA).

#### 2.3.2. Permeation Test

- Insert with 0.3 µm pore (Falcon/Corning, Cat# 353181, Durham, NC, USA).
- Pyrex 8 × 8 cloning cylinder (Corning, Cat# CLS31668, Glendale, AZ, USA).
- Nitrile O-ring 8 mm outer diameter (Lafco, Cat# LAF-6500-011, Montreal, QC, Canada).
- Twelve-well plate (Falcon, ThermoFischer, USA).
- Sterile gauze pads.
- Metal weight (Medical Grade 316, 2-B finish).
- Urea (ICN Biomedicals, Cat# 821527, Aurora, OH, USA).

#### 2.3.3. Urea Dosage

- Diacetylmonoxime (DAMO, C_4_H_7_NO_2_), Anhydrous Ethanol, (TCI AMERICA, Cat# B0683, Portland, OR, USA).
- Thiosemicarbazide (TSC, CH_5_N_3_S), Distilled Water, (Thermo Scientific, Cat# A14630.22, Ottawa, ON, USA).
- Ferric Solution (FeCl_3_·6H_2_O), Distilled Water, (Sigma-Aldrich, Cat# 157740, St. Louis, MO, USA).
- Concentrated Sulfuric Acid (98%, H_2_SO_4_), Distilled Water, (Sigma-Aldrich, Cat# 258105, St. Louis, MO, USA).
- Consumables: 2 mL Eppendorf tubes, tips, pipettes, 96-well microplate.

***OPTIONAL STEP*** for deproteinization:

- Trichloroacetic acid (TCA) (Fisher Scientific, Cat# A322-500, Fairlawn, NJ, USA).

### 2.4. Equipment

- Incubator 37 °C with 5% CO_2_.
- Safety cabinet for culture cell.
- Spectrophotometer: SpectraMax PLUS 384 (Molecular Devices, Sunnyvale, CA, USA) or other microplate reader reading at 520 nm.
- Water bath: set to 95 °C.
- Centrifuge: Micromax RF (Thermo IEC, Needham Heights, MA, USA) (or equivalent).

## 3. Procedure

### 3.1. Production of 3D Tissues

The self-assembly technique was used to produce tridimensional urological constructs as described previously [16]. A mix of cells was made based on this publication [17]. A second method based on collagen gels was made too. During the whole process cell culture medium needs to contain 50 µg/mL of 2-phospho-L ascorbic acid [18,19].

**Figure 1:**
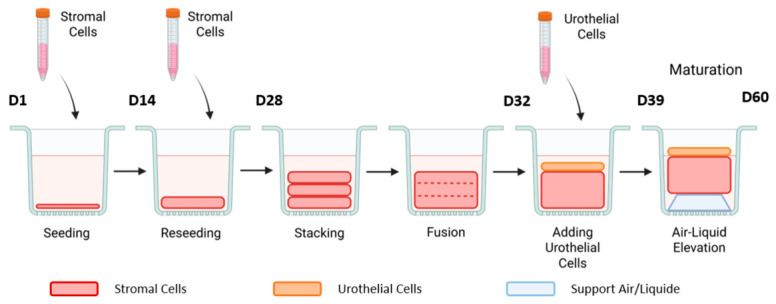
Schema of production of 3D tissues using the self-assembly method.

**Figure 2:**
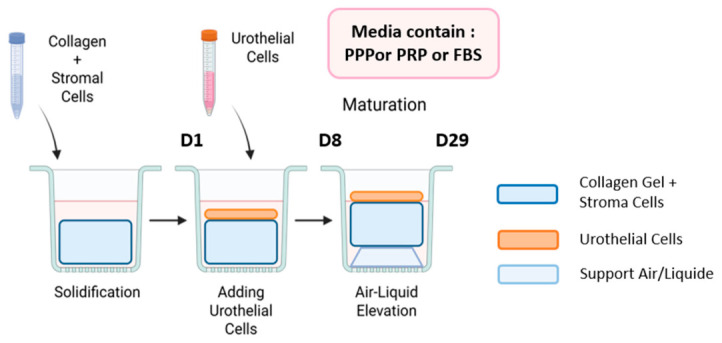
Schema of production of 3D tissues using collagen gels.

(a) Self-assembly tissues were produced, as shown in Figure 1.

1. Fibroblasts (UF or a mix BF: DF 80:20) at a density of 50,000 cells by cm^2^ were seeded into 6-well plates in Fb medium (DME supplemented with 10% Bovine Calf Serum, 100 U/mL penicillin and 25 mg/mL gentamicin).
2. A ring of Whatman 4 filter paper was placed as an anchorage paper on the bottom of the well and air/liquid support was placed on it to weigh the paper in the well.
3. The medium was changed every two days.
4. On day 14, we performed a second identical seeding step on the newly formed stromal sheets. Step 1 was then repeated.
5. We continued to change the media until day 28 to get enough neosynthesized extracellular matrix to obtain manipulable sheets.
6. We stacked three stromal sheets together in a 60 mm (cell culture untreated) Petri dish in a volume of 6 mL.
7. The day after, we completely removed the Fb medium and seeded UC at a density of 50,000 cells by cm^2^ on the 3D construction (UUC on UF-derived constructs and BUC on mix constructs). Maximum volume used for this seeding step was 200 µL in a 12-well plate.
8. This was incubated at 37 °C in 5% CO_2_ for 2–3 h.
9. We added 7 mL of UC medium (DMEM/Ham-F12 3:1 supplemented with 5% Fetal Clone II (FBS-H), 24.3 µg/mL adenine, 5 µg/mL crystallized bovine insulin, 1.1 µM hydrocortisone, 0.212 µg/mL isoproterenol hydrochloride, 10 ng/mL epidermal growth factor, 100 U/mL penicillin and 25 mg/mL gentamicin). The stack sheets were returned to the incubator.
10. After 1 week of culture, we raised the tissue to the air/liquid interface using a metallic support and continued to culture it for 21 additional days to induce urothelium differentiation.

(b) Collagen gels were produced, as shown in Figure 2.

1. We prepared a suspension solution of 0.5 million UF cells per mL in Fb medium.
2. We mixed a volume of 1.65 mL of this suspension with 1.65 mL of cold collagen in ice until a homogenous solution was obtained. A final volume of 3.3 mL allowed us to have the right working volume (3 mL), including losses due to collagen viscosity.
3. We cast one milliliter of the mixture in each of the 3 wells of a 12-well plate containing paper anchors (rings of Whatman 4 filter paper). This operation was repeated 12 times to obtain a total of 36 gels.
4. After gel solidification, we put metallic supports in place to prevent them from contracting.
5. We added one milliliter of serum-free UC medium containing 5% of FBS-H or poor-platelet plasma (PPP) or rich-platelet plasma (PRP) onto the gels (12 gels for each condition).
6. We changed the media three times a week, every 2 days.
7. After one-week, we seeded UUC at a density of 50,000 cells by cm^2^ onto the gels in 1 mL of UC medium containing 5% of FBS-H or PPP or PRP.
8. One week after seeding, we raised the gels to the air/liquid interface (using metallic supports) to allow the differentiation of the urothelial cells.
9. We changed the media every 2 days and continued for 21 additional days.
10. Three weeks later, the gels were ready for use.

### 3.2. Permeability Test

**Figure 3:**
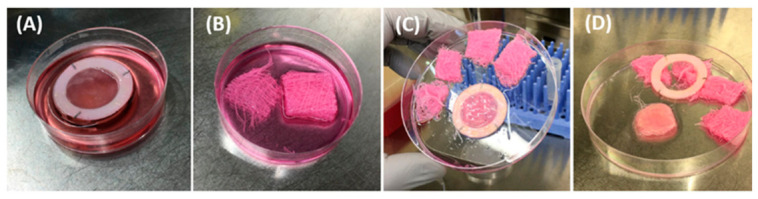
Samples preparation.

**Figure 4:**
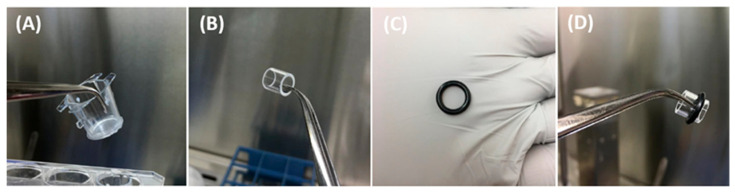
Donor chamber preparation.

**Figure 5:**
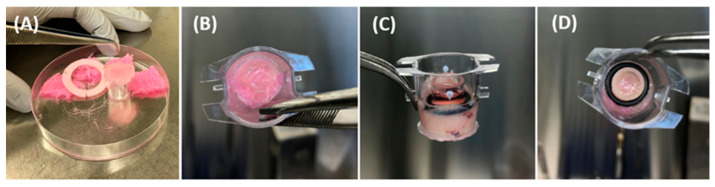
Assembly of the donor chamber.

**Figure 6:**
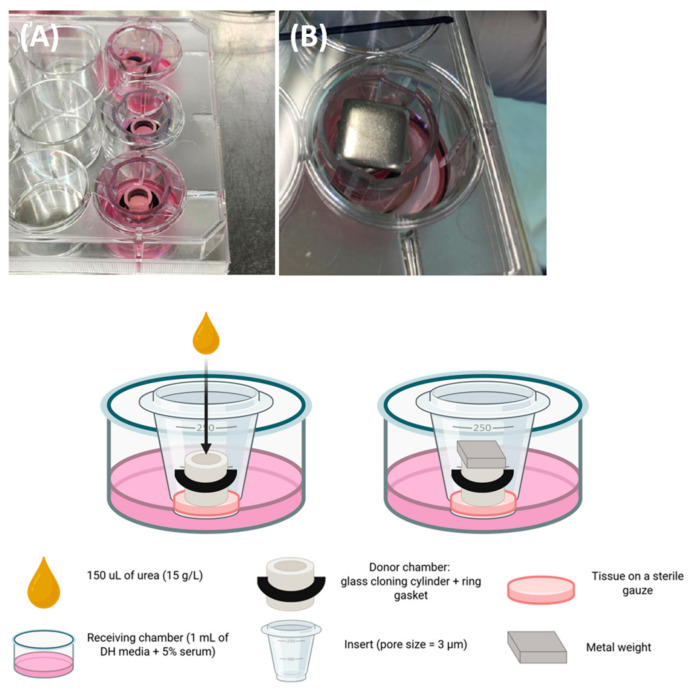
Installation into the receiver chamber and experimental setup.

The I-TEP test procedure is illustrated in Figure 3, Figure 4, Figure 5 and Figure 6.

I-TEP test procedure:

1. We placed the entire tissue sample (A) on a culture-medium-soaked gauze (B), forming a tissue–gauze construct (C).
2. We cut the tissue into a circular shape to separate it from the Whatman paper anchor (D).
3. In an insert with 3 µm pore membrane (A), we combined a cloning cylinder (B) with a surrounding O-ring (C) which led to this final construction (D). The O-ring stabilized the cylinder once it was positioned inside the insert and prevented lateral movement during assembly.
4. We placed the tissue–gauze construct (A) tightly at the bottom of a plastic insert containing a 3 µm pore membrane (B).
5. We inserted the cloning cylinder into the tissue to create a hermetic compartment (C-D).

**CRITICAL STEP:** The cylinder must be firmly pressed into the tissue already positioned inside the insert. To avoid damage to the porous membrane, the insert should be placed on a sterile flat surface (e.g., a sterile Petri dish) during this step.

6. We placed the assembled insert (donor chamber with tissue) into a well of a 12-well plate containing 1 mL of culture medium, forming the receiver chamber (A).
7. For the urea permeability experiments, we added 150 µL of a 15 g/L urea solution prepared in cell culture medium to the donor chamber; this concentration corresponded to the physiological one.
8. We put a metal weight on top of the assembly to hermetically seal the donor chamber, limit evaporation, and maintain constant pressure of the cloning cylinder against the tissue (B).
9. At 1, 2, 4, and 6 h, we collected receiver chamber media to quantify tissue permeability based on urea flux.
10. After each collection step, we added fresh culture medium in the receiver chamber; the volume of medium was always equal to the volume which was harvested.

### 3.3. Dosage of Urea (DAMO–TSC Method)

The assay utilized a calibration curve with urea standards ranging from 0 to 2 g/L (2, 1.5, 1, 0.75, 0.5, 0.25, 0 g/L) in the same solution as samples.

**Figure 7:**
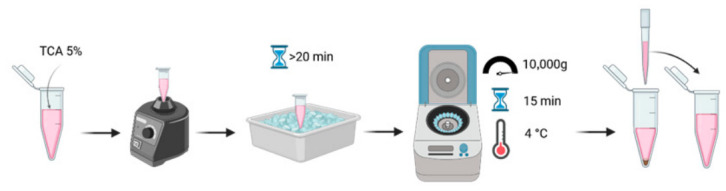
Schematic representation of sample deproteinization for serum/protein removal. Deproteination of samples was done using trichloroacetic acid (TCA) precipitation for a minimum of 20 min followed by a centrifugation to pellet the proteins.

***OPTIONAL STEP:*** Deproteinization of samples (required for samples containing proteins/serum) as shown in Figure 7.

1. We added trichloroacetic acid TCA 20% to the samples to reach a final concentration of 5% (41.7 µL into 125 µL).
2. This was gently shaken.
3. Then, it was incubated on ice for at least 20 min.
4. We then centrifuged it at 10,000× *g* (Relative Centrifuge Force, RCF) for 15 min at 4 °C.
5. We collected the supernatants in another clean Eppendorf.

#### Preparation of the Coloring Mixture

**CRITICAL STEP:** Prepare just before use:

The concentration of different solutions is detailed in Table S3.

**Figure 8:**
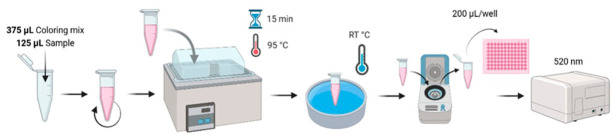
Schema of the DAMO–TSC dosage of urea.

- One volume of DAMO;
- One volume of TSC;
- Two volumes of ferric solution;
- Six volumes of 6 M sulfuric acid (H_2_SO_4_).
- Example for 10 mL: 1 mL DAMO, 1 mL TSC, 2 mL ferric, 6 mL H_2_SO_4_.
- The experimental protocol is shown in Figure 8.

1. In an Eppendorf tube, we added the following:
  - A measure of 125 µL (1/4) of sample or standard.
  - A measure of 375 µL (3/4) of coloring mixture, prepared just before use.
2. This mixture was gently shaken.
3. The mixture was heated in a water bath at 95 °C for 15 min.
4. Then, it was cooled to room temperature (RT ≈ 25 °C) in water, followed by a quick centrifugation to harvest the condensation water trapped in the cap.
5. We transferred 200 µL into a 96-well microplate in duplicate.
6. We measured the absorbance at 520 nm against a blank (reagents without urea).

## 4. Validation Methods

To validate the accuracy of I-TEP test, standard Franz diffusion cells were used in parallel to evaluate the permeability of similar reconstructed bladder tissues (BF/DF mix as stoma and BUC as epithelial cells) produced in the same batch [20,21].

Briefly, permeability studies were conducted using standard Franz diffusion cells comprising a donor and a receiver compartment, as previously described for engineered urological tissues. The effective diffusion area corresponded to 0.64 cm^2^. The engineered tissues were carefully positioned between the donor and receiver chambers, with the urothelium oriented toward the donor compartment. The receiver chamber was filled with 5 mL Fb medium and maintained at 37 °C (water-jacketed system) under continuous stirring using a magnetic stir bar to ensure homogeneous mixing. Urea was prepared at 15 g/L in DME and loaded into the donor compartment at the start of the experiment. At 1, 2, 4, 6 h time points, aliquots from the receiver compartment were collected to assess permeation, and the receiver solution was fully removed and replaced with fresh medium to maintain sink conditions.

The urea concentration in the samples was determined using a clinical-grade urea-nitrogen detector (Dimension Vista^®^, Siemens Healthcare Diagnostics, Tarrytown, NY, USA) or via DAMO–TSC technique.

## 5. Results

### 5.1. I-TEP Test Gave Comparable Results to Tests Done with Franz Diffusion Cell

**Figure 9:**
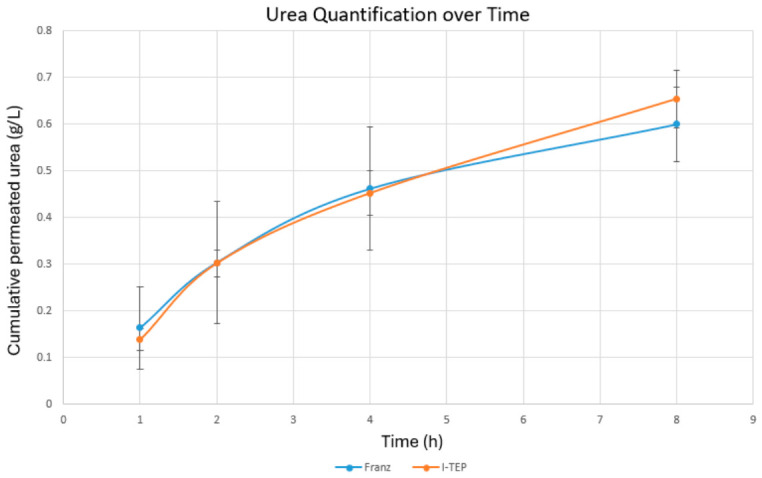
Comparison between Franz diffusion cells and I-TEP system. Quantification of urea after Franz diffusion cell (Franz) and I-TEP method (I-TEP) were presented. *N* = 3 for each technique. *p*-values are 0.85, 0.65, 0.58, and 0.43 for 1, 2, 4 and 8 h, respectively. The graph represents mean ± sd.

To validate the usefulness of the I-TEP system, it was essential to assess whether experiments performed using the I-TEP system yielded results comparable to those obtained with the Franz diffusion cell, which is considered the gold standard. To this end, identical tissue samples—bladder tissues reconstructed using the self-assembly method—were evaluated using both the Franz diffusion cell and the I-TEP system. Urea samples were sent to the hospital biochemistry laboratory for quantification, as described in the Validation Methods Section. As shown in Figure 9, a strong correlation was observed between the two techniques, with no significant differences between the conditions. The test has been performed on 3 samples, and will need more validation experiments if the results are to be completely affirmative. Nevertheless, these findings demonstrate that the I-TEP test can represent a reliable alternative to the gold-standard method.

### 5.2. Impact of Environmental Variables Such as Media Components

Tissue (native or reconstructed) must be incubated with various cell culture medium or solution to evaluate their permeability. DMEM:HamF-12 (UC medium) and Keratinocyte Serum-Free Medium (KSFM but containing 0.2% pituitary gland extract) were the most used in tissue engineering for cultivation of epithelial cells containing tissues. The DAMO–TSC dosage was tested for calibration curve in water (H_2_O), UC medium without serum and supplement (DH) or UC medium (DH5%DP). In the case of UC medium, a deproteinization step using TCA was done on the elements of the calibration curve.

**Figure 10:**
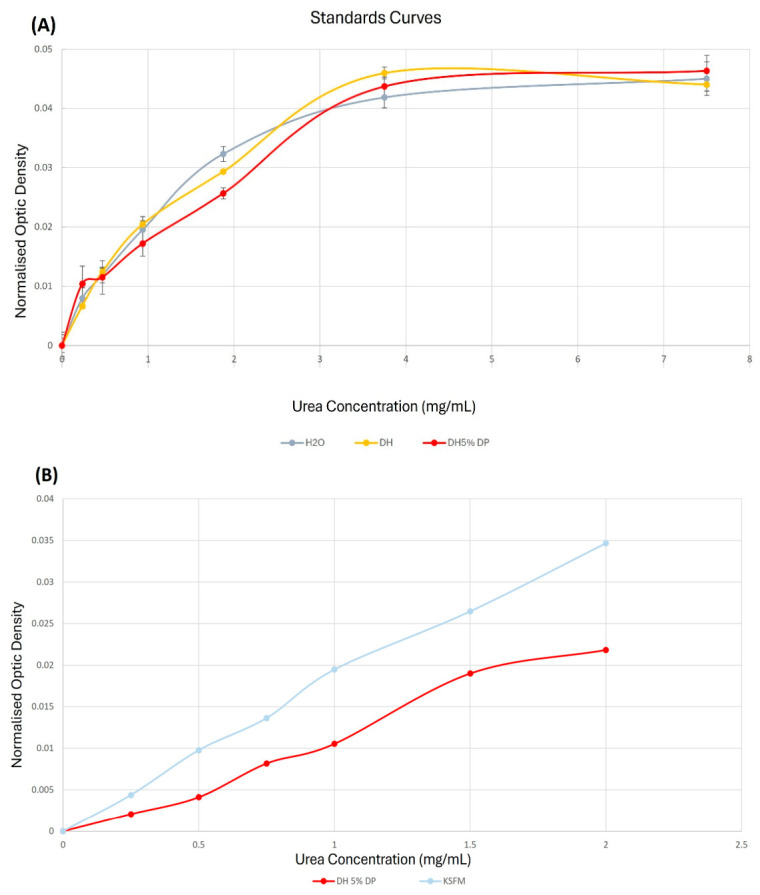
Calibration curve in different mediums. (**A**) Calibration curves made with water (H_2_O), DH only without serum (DH) and DH with 5% serum which was deproteinized (DH5% DP). Urea dilution starts at 7.5 mg/mL to 0 mg/mL. Each solution was duplicated. (**B**) Calibration curves in KSFM and DH 5% serum deproteinized. Concentration was made from 2 mg/mL to 0 mg/mL. SDs were negligible and are not represented.

Maximal concentration of calibration standards was 7.5 g/L: Figure 10A, indicates a 3 g/L urea concentration limit can been established leading to a signal saturation with higher concentrations. Nevertheless, using the I-TEP system the maximal concentration which can be reached is 0.15 mL × 15 mg/mL = 2.25 mg in 1.15 mL, i.e., 1.96 g/L. It is also clear that calibration curve done in solution containing no proteins gave the same profile. As the maximum urea concentration expected for standard curve is approximately 2 g/L. On Figure 10B, the DAMO–TSC dosage was carried out with KSFM and even if the profile is comparable, the absolute values were different resulting in different equations for the trend lines. The impact of the small amount of proteins into KSFM medium is then questioned, and more generally, the need to deproteinize the samples in medium containing proteins.

### 5.3. Deproteinization Is Required for Samples Containing Proteins

**Figure 11:**
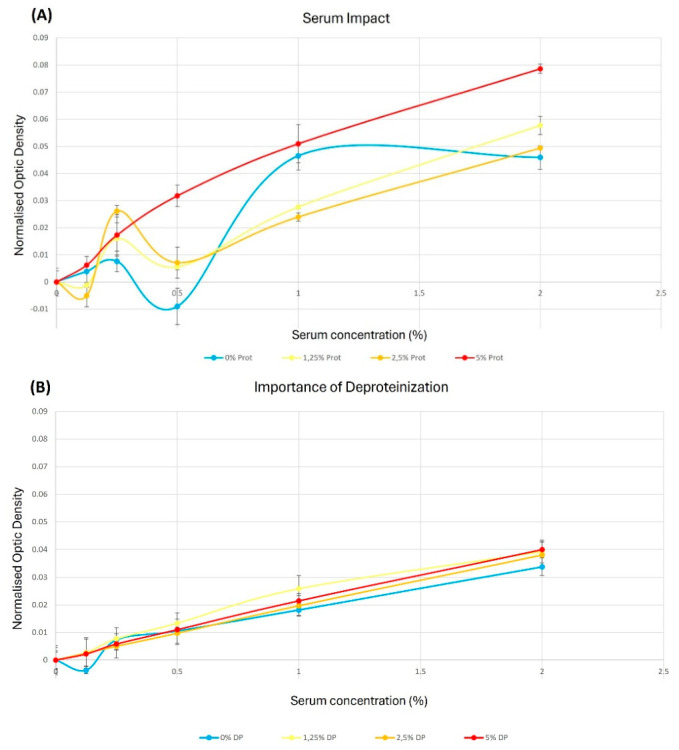

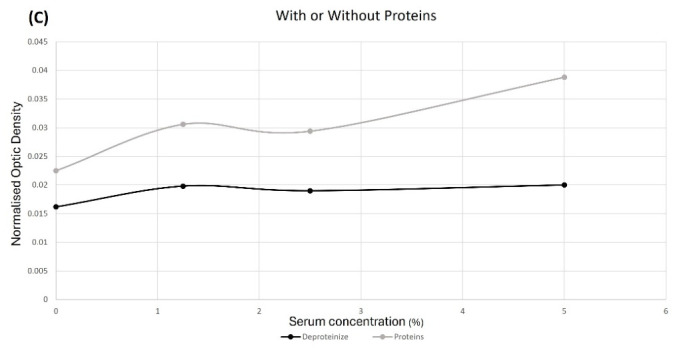
Comparison between untreated and deproteinized conditions. (**A**) Calibration curve using standard in medium with 5% (5% Prot), 2.5% (2.5% Prot), or 1.25% of serum (1.25% Prot) or without serum (0% Prot) were tested in a range of concentration between 0 mg/mL to 2 mg/mL urea. (**B**) Calibration curve using standard in medium with 5% (5% DP), 2.5% (2.5% DP), or 1.25% of serum and deproteinized (1.25% DP) or without serum but treated as the deproteinized standards (0% DP) were tested in a range of concentration between 0 mg/mL to 2 mg/mL urea. (**C**) Slope of the calibration curve in untreated condition (proteins, grey line) or conditions after deproteinization protocol (deproteinized, black line). SDs were negligible and are not represented.

In the literature, deproteinization is required for some samples due to the presence of serum, a protein-rich mixture, which could interfere with DAMO–TSC dosage [22]. To verify this statement, untreated and deproteinized samples were compared. Whereas curves present a low linearity for untreated samples with R^2^ ranging from 0.693 to 0.9632 (Figure 11A), deproteinized conditions (Figure 11B) showed robust trend lines with R^2^ ranging from 0.9481 to 0.9997. Furthermore, Figure 11C, representing the slope of the calibration curves, showed that, without deproteinization, the quantification increased with the serum concentration in the media, yielding up to twice the concentration observed in deproteinized condition. Taken altogether, these findings confirm that deproteinization is a mandatory step for accurate quantification.

### 5.4. Cryopreservation of the Urea

**Figure 12:**
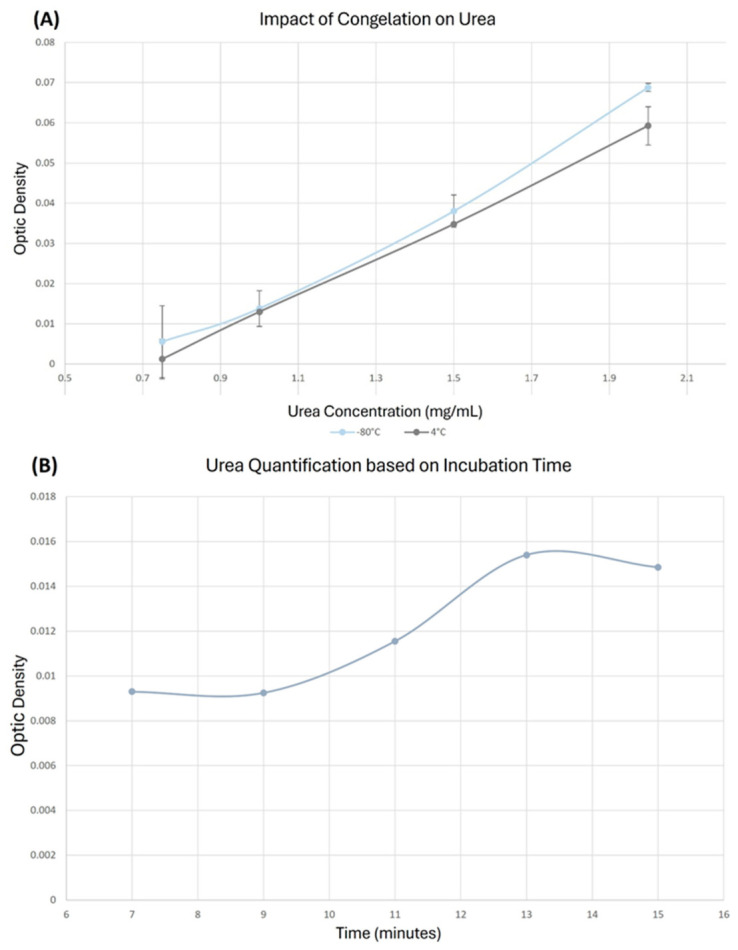
Impact of storage conditions and duration at 95 °C incubation. (**A**) Comparison between solution store in −80 °C for 4 months (Blue) and a fresh solution (Grey) of urea at 15 mg/mL. *n* = 2, *p*-values were 0.885; 0.590; 0.991; 0.033 for 0.1175, 1, 1.5, 2 g/L urea. (**B**) Dosage of urea after different incubation time at 95 °C from 5 min to 15 min. SDs were negligible and are not represented.

To optimize the test, the cryopreservation of urea standards at −80 °C was tested in comparison to freshly prepared standards. It allows us to determine whether a potential degradation of urea during storage can affect the results. Comparison of two standard curves (Figure 12A) showed no significant difference between fresh and frozen urea, except at higher urea concentration. The slope of the curve is also slightly different (0.0461 with R^2^ of 0.9994 vs. 0.509 with a R^2^ of 0.9898 for fresh and frozen solutions, respectively). These results suggest that storage conditions have a limited impact, but if researchers want to store their calibration curves, they need to adjust the values even if it is better for accuracy of the dosage to use always calibration curves prepared freshly in the same solutions as the samples. 

### 5.5. Incubation at 95 °C for 13 or 15 Minutes Produced a Higher Signal

Additionally, while the time incubation test determined a 15 min incubation at 95 °C as standard time, some studies mentioned that a shorter time of 9 min is sufficient [11]. Duration of incubation at 95 °C was evaluated in Figure 12B. Results clearly indicated that 13 to 15 min was the optimal time of incubation, the signal reaching a plateau at 13 min. Therefore, a 15 min incubation period was selected to ensure maximum accuracy and minimize variability.

### 5.6. Validation with Biological Samples

**Figure 13:**
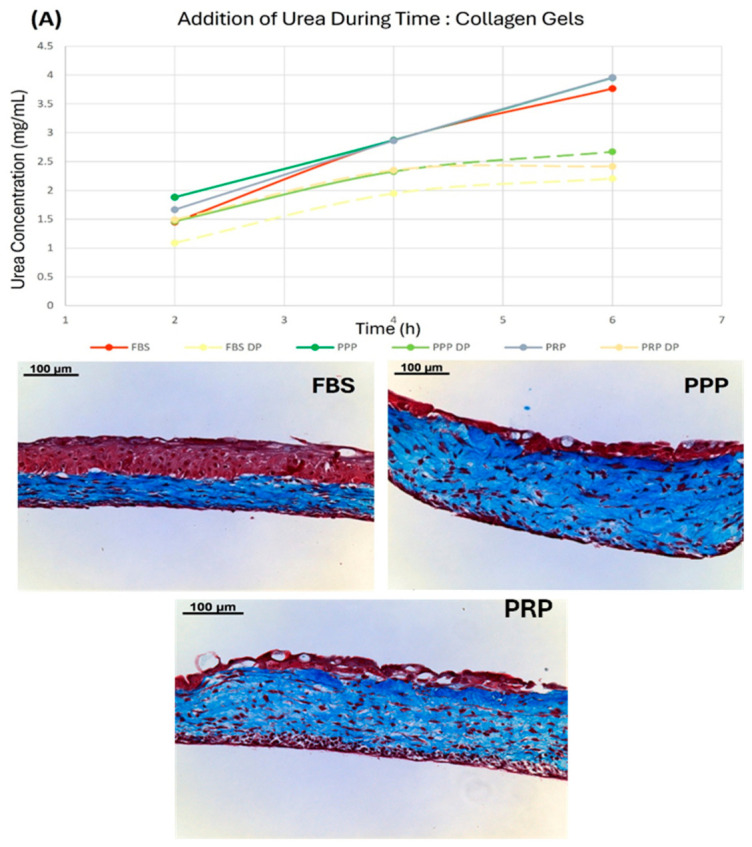

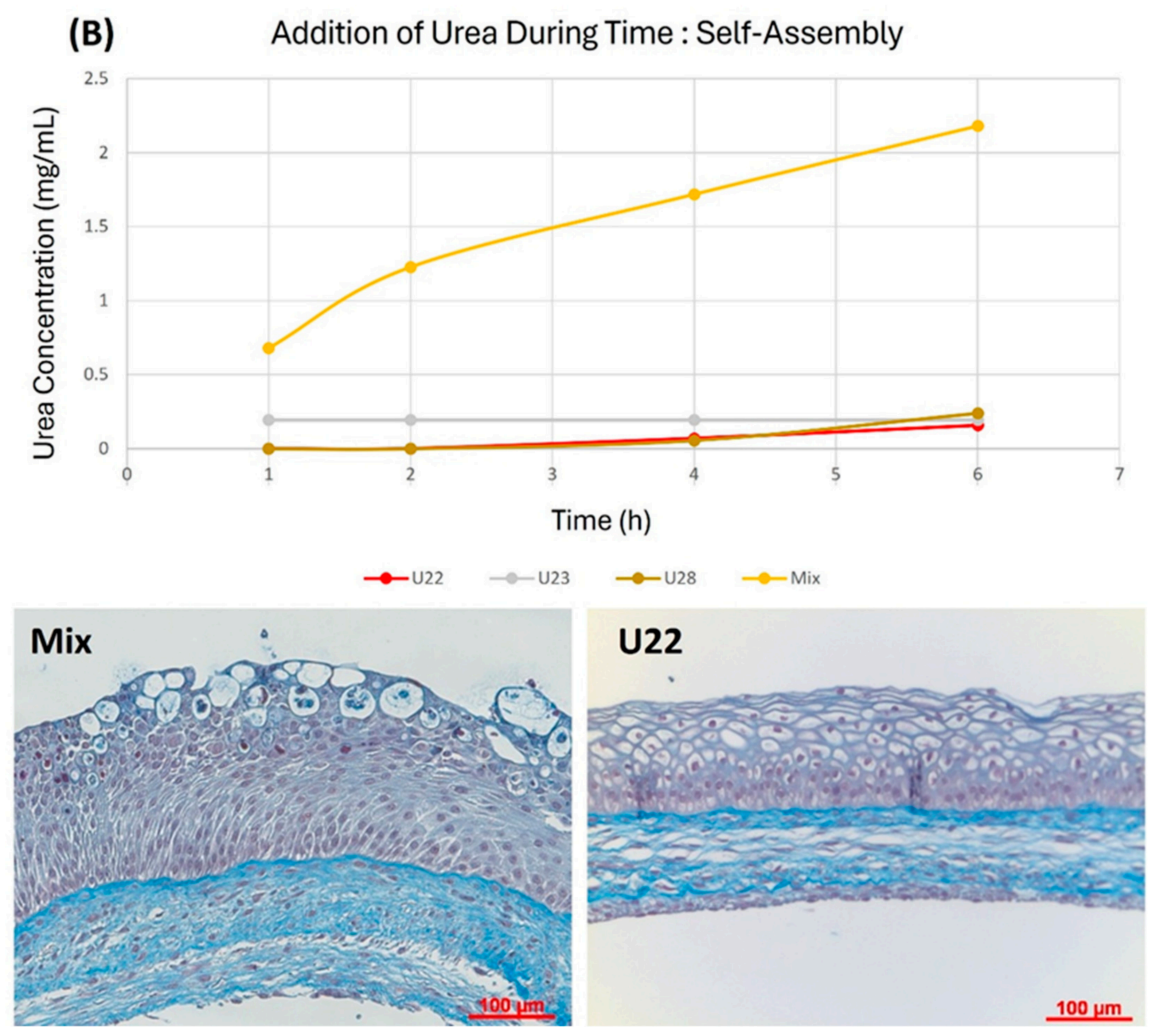
Permeability correlation. (**A**) DAMO–TSC test on sample from different collagen gel with or without deproteinization. Conditions are collagen gel with Fetal Clone II (FBS), deproteinized Fetal Clone II (FBS DP), collagen gel with poor platelet plasma (PPP), deproteinized PPP gel (PP DP), collagen gel with rich platelet plasma (PRP) and PRP gel deproteinized (PRP DP). All media used is UC medium. *N* = 3. SDs were negligible and are not represented. Masson’s trichrome staining was performed on slices of PBS, PPP and PRP supplemented tissues. (**B**) Urethral tissues reconstructed using cells (UF and UUC) from different donors, 22 years old (U22), 23 years old (U23), 28 years old (U28) and a mix of 80/20% of BF and DF and BUC (Mix). Masson’s trichrome staining was performed on slices of tissue for Mix and U22, U23 and U28 (only U22 was presented due to very high similarities of all urethral tissues).

To conduct in-depth testing on real tissue samples, I-TEP test was performed on tissues produced by two different operators and using different tissue engineering techniques: collagen hydrogels, a simple technique, and self-assembly, a more complex technique with more refined extracellular matrix elements. Firstly, test was carried out with collagen-based tissues (Figure 13A). The disparity between deproteinized and non-deproteinized samples was obvious. Non-deproteinized samples overpassed the physical limit of 2 g/L to reach 4 g/L. In contrast, it was not the case for deproteinized samples, showing the beginning of stagnation curve as soon as they reach 2 g/L, indicating that the maximum has been reached.

Interestingly, whereas no difference can be observed without deproteinization, the treated conditions showed a difference between FBS on one side and PPP and PRP on the other side. The permeability of the FBS-H condition was 95% of that of the PPP condition in the untreated condition, whereas it was about 82% of the PPP deproteinized condition. These observations correlated with the histological analysis. The greatest permeability values were obtained in PPP and PRP conditions, where the urothelium is impermeable, whereas gels cultivated with FBS-H were a little less permeable.

Similar results were observed with the tissue produced by the self-assembly technique (Figure 13B). Constructs composed of 100% urethral cells showed near zero permeability over time, contrarily to mixed-cell tissues where permeability is higher. These data are consistent with histological observations. While the tissue “Mix” presents a lot of secretory cells at their surface which reduce the surface of the uroplakin plaque (the plaque protecting the urothelium from urea leakage), urethral tissue presents a more homogenous epithelium.

## 6. Discussion

The primary objective of this study was to validate the I-TEP system as a viable alternative to the Franz diffusion cell system. Our results confirm a high degree of correlation between the two systems (Figure 9).

However, there are few limits that need to be optimized. Firstly, the small volume of the insert may hinder sample insertion into it for some users, particularly when the tissue to be tested is adhesive, for instance, in the presence of abundant mucus.

Moreover, depending on the molecule being tested—for instance, when assessing alternative penetration pathways across an epithelium—diffusion into the receiver chamber could be enhanced by agitation. For example, a micro-magnetic stir bar could be placed in the well (receiver compartment), and the 12-well plate positioned on a magnetic stirrer.

To continue with the disadvantages, it should also be noted that DAMO–TSC dosing requires the addition of an extra step, namely deproteinization, as shown in Figure 11, when serum-supplemented media are used. This step adds complexity and a potential source of human error to the workflow, but it is the only way to ensure the accuracy required for sensitivity at micromolar levels. For future iterations, the development of a standardized deproteinization kit or an automated centrifugal filtration step could further improve this process.

Note that DAMO–TSC can be sensitive to various experimental conditions, and some heterogeneity can appear between dosage results in several iterations, so it is better to compare results in the same dosage or to be able to have samples which allow comparison [11,23].

Changing the cell culture media before starting the I-TEP experiment for protein-free medium can be another solution because incubation time is short (no more than 24 h).

In the present study, epithelial permeability was assessed using urea as a reference molecule, providing an initial evaluation of barrier function. While urea is a well-established small, hydrophilic marker of epithelial permeability, additional probe molecules with distinct physicochemical properties could further strengthen the validation of the model. In particular, the combined use of hydrophilic and lipophilic compounds—such as caffeine and testosterone, respectively—is commonly employed to discriminate between paracellular and transcellular transport pathways across epithelial barriers [24]. Such complementary permeability assays would allow a more comprehensive characterization of the barrier properties of the reconstructed tissue and help refine the interpretation of transport mechanisms under different experimental conditions.

It could be also noted that the experiment has been done with an average of 3 replicates and validation should be performed to confirm those results.

There are positive aspects and advantages to using this technique. The most important aspect brought by I-TEP system is its low-cost profile. For less than USD 30, a lab can obtain the basics for performing the test, with further expenses comprising USD 82 for every 12 samples tested. Compared to the cost of a Franz Diffusion cell system, I-TEP remain competitive until the 43rd assay of 12 samples, i.e., 500 samples (Table 1 and Table 2).

This cost analysis shows that more than **42 experimental rounds of 12 samples** (i.e., over 500 samples) would be required using a Franz diffusion cell setup to reach a cost comparable to that of the I-TEP system. The analysis indicates that sporadic use (≤500 samples) favors the I-TEP system, whereas extensive and long-term use makes the Franz diffusion cell approach more cost-effective. This analysis does not account for material loss or breakage associated with the Franz diffusion cell technique, which typically relies on glass components.

Secondly, through the association with DAMO–TSC, research teams become completely autonomous and no longer depend on external third parties such as clinical biochemistry labs. DAMO–TSC allows reducing waiting time, especially time to obtain results due to the shipment of samples. Moreover, the DAMO–TSC technique costs only USD 4 for every 100 samples, whereas the urease kit costs USD 538 for the same number of samples, meaning it is 133 times more expensive.

In conclusion, the I-TEP system democratizes the permeability test for smaller research units. Therefore, the system’s compatibility with standard well-plates allows for high-throughput screening of 3D tissues without specialized equipment beyond a standard incubator and spectrophotometer. This system bridges the gap between high-tech “Organ-on-a-Chip” systems and traditional methods, offering a practical solution for the routine assessment of 3D tissue barrier function.

Beyond the specific findings of this study, our group has pursued, over the past decade, a cross-cutting objective of developing easy-to-implement, low-cost and accessible protocols for advanced tissue engineering and functional testing (e.g., [15]). By simplifying the key steps in tissue reconstruction and designing functional assays requiring minimal equipment, we aim to reduce technical and financial barriers without compromising scientific robustness. The permeability assay presented here fully aligns with this philosophy of accessibility and reproducibility.

## Figures and Tables

**Table 1:** Cost analysis comparing the Franz diffusion cell and the I-TEP system.

Franz Diffusion Cell	Price (USD)	I-TEP Test	Price (USD)
12 cell-model reusables after rinsing	3510	Cylinder × 12	20
		O-ring × 12	6.5
		Cover × 12	0.9
Experimental cost (broken material)	variable	Experimental cost (12-weel insert × 12)	82

**Table 2:** Cost comparison between DAMO–TSC-based urea quantification and a commercial urease kit.

Reagent	Weight (g)	Price (USD)	Stock Concentration	Price/1 mL (USD)	Volume/Sample (mL)	Price/Sample (USD)
DAM	25	96	0.2%	0.00768		
TSC	100	40	0.05%	0.0002	0.0375	0.0000075
FeCl_3_	100	58	0.05 M	0.0047038	0.075	0.000352785
H_2_SO_4_	100	43	6 M	0.1419	0.225	0.0319275
EtOH	1000	120	100%	0.12		
DAM in EtOH				0.12768	0.0375	0.004788
TCA	250	131	20%	0.1048	0.03125	0.003275

## Data Availability

Data are available upon request to the corresponding authors.

## Supplementary Materials

The following supporting information can be downloaded at: https://www.mdpi.com/article/10.3390/mps9030073/s1, Table S1: Fb medium; Table S2: UC medium; Table S3: Material used for Dosage of urea.

## References

[1] Dalghi M.G., Montalbetti N., Carattino M.D., Apodaca G. (2020). The Urothelium: Life in a Liquid Environment. Physiol. Rev..

[2] Song J., Xu Z., Xie L., Shen J. (2025). Recent Advances in Studying In Vitro Drug Permeation Across Mucosal Membranes. Pharmaceutics.

[3] Supe S., Takudage P. (2021). Methods for evaluating penetration of drug into the skin: A review. Ski. Res. Technol..

[4] Gu Y., Zhang L., Du Z., Wang C., Deng W., Liu Y.U., Tang B. (2026). Aging of the Blood-Brain Barrier and Altered Permeability to Peripheral Immune Cells: Implications for Central Nervous System Disorders. Aging Dis..

[5] Zupančič D., Romih R. (2021). Immunohistochemistry as a paramount tool in research of normal urothelium, bladder cancer and bladder pain syndrome. Eur. J. Histochem..

[6] Bittermann K., Goss K.U. (2017). Predicting apparent passive permeability of Caco-2 and MDCK cell-monolayers: A mechanistic model. PLoS ONE.

[7] Al-Awqati Q. (1999). One hundred years of membrane permeability: Does Overton still rule?. Nat. Cell Biol..

[8] Ussing H.H., Zerahn K. (1951). Active transport of sodium as the source of electric current in the short-circuited isolated frog skin. Acta Physiol. Scand..

[9] Franz T.J. (1975). Percutaneous absorption on the relevance of in vitro data. J. Investig. Dermatol..

[10] Intarakumhaeng R., Alsheddi L., Wanasathop A., Shi Z., Li S.K. (2019). Skin Permeation of Urea Under Finite Dose Condition. J. Pharm. Sci..

[11] Evans R.T. (1968). Manual and automated methods for measuring urea based on a modification of its reaction with diacetyl monoxime and thiosemicarbazide. J. Clin. Pathol..

[12] Vurek G.G., Knepper M.A. (1982). A colorimeter for measurement of picomole quantities of urea. Kidney Int..

[13] Rahmatullah M., Boyde T.R. (1980). Improvements in the determination of urea using diacetyl monoxime; methods with and without deproteinisation. Clin. Chim. Acta.

[14] Langenfeld N.J., Payne L.E., Bugbee B. (2021). Colorimetric determination of urea using diacetyl monoxime with strong acids. PLoS ONE.

[15] Brownell D., Elia E., Pellerin F.A., Chabaud S., Larochelle S., Moulin V.J., Laungani A., Bolduc S. (2025). Isolation and characterization of epithelial cells and fibroblasts from the human penile urethra. Front. Bioeng. Biotechnol..

[16] Chabaud S., Rousseau A., Marcoux T.L., Bolduc S. (2017). Inexpensive production of near-native engineered stromas. J. Tissue Eng. Regen. Med..

[17] Caneparo C., Chabaud S., Fradette J., Bolduc S. (2022). Engineered human organ-specific urethra as a functional substitute. Sci. Rep..

[18] Saba I., Jakubowska W., Bolduc S., Chabaud S. (2018). Engineering Tissues without the Use of a Synthetic Scaffold: A Twenty-Year History of the Self-Assembly Method. BioMed Res. Int..

[19] Pellerin F.A., Dufresne É., Chabaud S., Orabi H., Bolduc S. (2024). Mimicking Urinary Tract Infections Caused by Uropathogenic Escherichia coli Using a Human Three-Dimensional Tissue Engineering Model. Microorganisms.

[20] Tremblay A., Simard M., Morin S., Pouliot R. (2021). Docosahexaenoic Acid Modulates Paracellular Absorption of Testosterone and Claudin-1 Expression in a Tissue-Engineered Skin Model. Int. J. Mol. Sci..

[21] Simard M., Julien P., Fradette J., Pouliot R. (2019). Modulation of the Lipid Profile of Reconstructed Skin Substitutes after Essential Fatty Acid Supplementation Affects Testosterone Permeability. Cells.

[22] Dawson R.M. (1993). The diacetylmonoxime assay of urea, its application to the assay of diacetylmonoxime and a comparison with other methods for the analysis of diacetylmonoxime. J. Appl. Toxicol..

[23] Chen S., Lin S., Ding L.X., Wang H. (2023). Modified Diacetylmonoxime-Thiosemicarbazide Detection Protocol for Accurate Quantification of Urea. Small Methods.

[24] Kurtz S.L., Lawson L.B. (2018). Determination of permeation pathways of hydrophilic or hydrophobic dyes through the mammary papilla. Int. J. Pharm..

