# Supplementary material for: I-TEP: A Simple and Affordable Method to Measure Permeability in Reconstructed Tissues Combined with DAMO–TSC-Based Urea Assay
Source: Methods Protoc. 2026 May 3;9(3):73. doi: 10.3390/mps9030073 (PMC13214736; doi:10.3390/mps9030073)
Supplement: Supplementary file 1 [file mps-09-00073-s001.zip › mps-4202597-supplementary.pdf]

Supplementary materials:

Table S1. Fb medium.

Reagent	Concentration	Product reference
Dulbecco-Vogt modification of Eagle's medium	**90%** of the total volume	Gibco, Cat# 12800-017, Grand Island, NY, USA
Bovine Calf Serum (FBS)	**10%** of the total volume	Corning, Cat# 35-053-CM, Woodland, CA
Penicillin	100U/mL	Sigma-Aldrich, Cat# P3032, St. Louis, MO, USA
Gentamicin	25mg/mL	MP Biomedicals, Cat# 190057, Solon, OM, USA

Table S2. UC medium.

Reagent	Concentration	Product reference
Dulbecco-Vogt modification of Eagle's medium	**71.25%** of the total volume	Gibco, Cat# 12800-017, Grand Island, NY, USA
Ham's F12	**23.75%** of the total volume	Gibco, Cat# 21700-075, Grand Island, NY, USA
Fetal Clone II (FBS-H)	**5%** of the total volume	Hyclone Cytiva Fetal clone II, Cat# SH3066.03, Logan, UT, USA
Adenine	24.3 μg/mL	Sigma-Aldrich, Cat# A2786, St. Louis, MO, USA
Crystallized bovine insulin	5 μg/mL	Sigma-Aldrich, Cat# I5500, St. Louis, MO, USA
Hydrocortisone	1.1 μM	Galenova, Cat# HY220-0005, Scarborough, Canada
Isoproterenol hydrochloride	0.212 μg/mL	Sigma-Aldrich, Cat# I5627, St. Louis, MO, USA
Epidermal growth factor	10 ng/mL	Austral Biologicals, Cat# GF-010-8, San Ramon, CA, USA
Penicillin	100U/mL	Sigma-Aldrich, Cat# P3032, St. Louis, MO, USA
Gentamicin	25 mg/mL	MP Biomedicals, Cat# 190057, Solon, OM, USA

Table S3. Material used for Dosage of urea.

Reagent	Concentration	Solvent	Product reference
Diacetylmonoxime (DAMO, $C_4H_7NO_2$)	0.2% v/w or 20mM	Anhydrous Ethanol	TCI AMERICA, Cat# B0683, Portland, OR
Thiosemicarbazide (TSC, CH_5N_3S)	0.05% v/w or 5.5mM	Distilled Water	Thermo Scientific, Cat# A14630.22, Ottawa, ON

Ferric Solution ($FeCl_3 \cdot 6H_2O$)	0.81% v/w or 0.05M	Distilled Water	Sigma-Aldrich, Cat# 157740, St Louis, MO
Concentrated Sulfuric Acid (98%, H_2SO_4)	~33% v/v or 6M	Distilled Water	Sigma-Aldrich, Cat# 258105, St Louis, MO
